# INCARCERATION AS A RISK FACTOR FOR IMPAIRMENT IN COGNITIVE FUNCTIONING

**DOI:** 10.1093/geroni/igad104.3427

**Published:** 2023-12-21

**Authors:** George Daniel, Cedric Williams, Joel Wilkinson, Denee Mwendwa

**Affiliations:** Howard University, Washington, District of Columbia, United States; Howard University, Washington, District of Columbia, United States; Howard University, Washington, District of Columbia, United States; Howard University, Washington, District of Columbia, United States

## Abstract

**Background:**

Incarceration is a social determinant of health with public health implications, especially among minoritized communities of color. The experiences of incarcerated people have deleterious implications for mental and physical health outcomes. Research has demonstrated an association between incarceration and poorer global cognition in older adults. The current study examines the long-term impact of incarceration on cognitive functioning in older adults and examines whether this relationship is moderated by race, ethnicity, and sex.

**Methods:**

Data were collected on participants (n=7,557) in the Health and Retirement Study (HRS). They completed a background questionnaire, psychosocial assessments, and a series of neuropsychological tests. T-tests and Chi-Squared analyses determined differences in demographic and health variables by incarceration status. Linear regression analysis determined if incarceration status predicts poorer cognitive functioning in older adults while controlling for age, race, sex, ethnicity, education, hypertension, diabetes status, wealth, depression, and stroke. Subgroup analyses determined whether associations were modified by race, ethnicity, or sex.

**Results:**

Our sample is on average 74 years old, 13% Black, and 60% female. Formerly incarcerated people were younger, disproportionately Black, less wealthy and educated, and more unhealthy. Analyses indicated that incarceration predicted reduced performance in delayed verbal recall (B = -0.19, p = 0.047) and this relationship may be stronger in Non-Hispanics, African-Americans and Women.

**Conclusion:**

Incarceration may negatively impact long-term memory in older adults and varies by gender, race, and ethnicity. Future studies should investigate variables that influence this relationship, including SES, depression, and chronic illnesses.

